# Serum trimethylamine-N-oxide is associated with incident type 2 diabetes in middle-aged and older adults: a prospective cohort study

**DOI:** 10.1186/s12967-022-03581-7

**Published:** 2022-08-18

**Authors:** Shu-yi Li, Si Chen, Xiao-ting Lu, Ai-ping Fang, Yu-ming Chen, Rong-zhu Huang, Xin-lei Lin, Zi-hui Huang, Jing-fei Ma, Bi-xia Huang, Hui-lian Zhu

**Affiliations:** 1 Department of Nutrition, School of Public Health, Sun Yat-Sen University, Guangzhou, 510080 People’s Republic of China; 2 Department of Medical Statistics & Epidemiology, School of Public Health, Sun Yat-Sen University, Guangzhou, People’s Republic of China; 3 Guangdong Provincial Key Laboratory of Food, Nutrition and Health, School of Public Health, Sun Yat-Sen University, Guangzhou, People’s Republic of China

**Keywords:** Trimethylamine-N-oxide, Metabolites, Type 2 diabetes, Middle-aged and older adults, Prospective cohort

## Abstract

**Background:**

The role of trimethylamine-N-oxide (TMAO) in the development of diabetes remains controversial, and prospective data are few. We aimed to investigate the association between serum TMAO and incident type 2 diabetes in middle-aged and older adults.

**Methods:**

This study was based on the Guangzhou Nutrition and Health Study (GNHS), a community-based prospective cohort study in China. A total of 2088 diabetes-free participants aged 40–75 years were included from 2008 to 2010. Incident type 2 diabetes was ascertained during follow-up visits. Baseline serum TMAO was measured by high-performance liquid chromatography with online electrospray ionization tandem mass spectrometry. Hazard ratios (HRs) and 95% confidence intervals (95% CIs) for diabetes across tertiles of serum TMAO were calculated using Cox proportional hazard models. Prospective associations of serum TMAO with changes in glycemic traits (fasting glucose, HbA1c, insulin, HOMA-IR) over time were estimated using linear mixed-effects models (LMEMs).

**Results:**

We ascertained 254 incident type 2 diabetes cases during a median follow-up of 8.9 years. The median (interquartile range) of serum TMAO was 1.54 (0.86–2.91) μmol/L. From the first to the third tertile of serum TMAO, the multivariable-adjusted HRs for diabetes were 1.00 (reference), 1.17 (95% CI: 0.84–1.61), and 1.42 (95% CI: 1.03–1.96) (*P*-trend = 0.031). LMEMs showed that the estimated yearly change in fasting glucose was 0.011 (0.001–0.022) mmol/L/y in the highest tertile of serum TMAO, compared with the lowest tertile (*P*-interaction = 0.044). Serum TMAO was not associated with longitudinal changes in HbA1c, insulin or HOMA-IR.

**Conclusions:**

Our findings suggested that higher serum TMAO was associated with a higher risk of type 2 diabetes and an increase in fasting glucose among middle-aged and older Chinese adults.

*Trial registration:* NCT03179657. https://clinicaltrials.gov/ct2/show/NCT03179657?term=NCT03179657&draw=2&rank=1

**Supplementary Information:**

The online version contains supplementary material available at 10.1186/s12967-022-03581-7.

## Background

Diabetes has become a critical public health problem. The number of adults with diabetes had risen to 537 million globally in 2021 [1]. China is experiencing an unprecedented epidemic of diabetes, with a prevalence increased from 0.67% in 1980 to 12.4% in 2018 [2]. Patients with type 2 diabetes, the most common type of diabetes, have high cardiovascular risk [3]. Cardiovascular disease is the major complication in diabetes and the leading cause of mortality in patients with diabetes [4–7].

Lifestyle factors contribute to the onset and progression of type 2 diabetes, and studies have suggested that diet and gut microbiota may impact type 2 diabetes [8]. Trimethylamine-N-oxide (TMAO) is found naturally in marine fish, and TMAO is also generated from animal- or plant-source foods containing choline, betaine, or carnitine [9]. Dietary choline, betaine and carnitine are converted into trimethylamine (TMA) by gut microbiota. TMA is subsequently oxidized to TMAO by flavin-containing monooxygenase-3 (FMO3) in the liver [10]. Recent animal studies have suggested that TMAO plays a role in glucose and lipid homeostasis, which may cause impaired glucose tolerance, insulin resistance, oxidative stress in adipose tissue [11, 12]. TMAO may be a potential link between diet, gut microbiota, and lifestyle-related diseases [13]. Considerable evidence from epidemiology studies has demonstrated that higher circulating TMAO levels are associated with increased risks of obesity [14], non-alcoholic fatty liver [15], and cardiovascular disease [16] in adults. On one hand, obesity, inflammation and other cardiovascular risk factors play important roles in the development of type 2 diabetes [17–20]; on the other hand, type 2 diabetes shares many common risk factors or underlying mechanisms with the above-mentioned lifestyle-related diseases, and thus elevated TMAO levels may also increase the risk of type 2 diabetes.

A recent meta-analysis suggests a positive dose-dependent association between circulating TMAO levels and increased type 2 diabetes risk [21]. Due to limited reports, most of studies included in the present meta-analysis examine the association between TMAO and cardiovascular disease or enrolled participants with a high cardiovascular risk, but few studies were designed for diabetes. Therefore, a series of cardiovascular-related indicators may lead to heterogeneity. Although cross-sectional studies and case–control studies have found higher circulating TMAO levels was associated with increased diabetes risk [22, 23], a prospective study of older US adults from the Cardiovascular Health Study did not suggest a positive association [24]. Longitudinal observational studies originally investigating the association between circulating TMAO and diabetes risk were scarce and have shown inconsistent results. A case-cohort study among elderly Mediterranean individuals reported an inverse association [25]; a prospective cohort study among Norwegian patients with suspected stable angina pectoris reported no association [26]. Differences in dietary pattern and genetic variation among study population from different regions may also be the underlying source of inconsistent result. In these studies, most of the participants were from the United States and Europe, while few studies were conducted in Asian [27, 28].

Therefore, in this community-based prospective cohort study with a median follow-up of 8.9 years, we aimed to examine the temporal relationship and the magnitude of association between serum TMAO and (1) the incidence of type 2 diabetes; (2) the yearly changes in glycemic traits, including fasting glucose, glycated hemoglobin (HbA1c), insulin, and homeostasis model assessment of insulin resistance (HOMA-IR) in southern China.

## Methods

### Study design

Data for this study were from the Guangzhou Nutrition and Health Study (GNHS), an ongoing community-based prospective cohort study. Between July 2008 and June 2010, we included 3169 participants, living in Guangzhou city (South China) for more than five years, aged 40–75 years. A questionnaire survey, anthropometric measurements, blood pressure measurement and fasting blood collection were conducted at baseline and every three years, and three follow-up visits were conducted up to March 2021. The study protocol of GNHS was registered in ClinicalTrials.gov as NCT03179657 and was approved by the Ethical Committee of the School of Public Health at Sun Yat-sen University. Written informed consent was obtained from each participant.

We excluded participants according to our prespecified criteria: (1) diabetes at baseline (*n* = 197); (2) self-report malignant tumor (*n* = 10), chronic renal failure (*n* = 6), stroke (n = 28) or myocardial infarction (*n* = 20) at baseline; (3) without measurements of TMAO (*n* = 196) or fasting glucose (*n* = 150); (4) those with extremely high or low energy intake (> 4000 kcal/d or < 800 kcal/d for men; > 3500 kcal/d or < 500 kcal/d for women) (*n* = 37); (5) missing data on diet variables or other variables (e.g., socio-demographics) (*n* = 75); (6) without any follow-up data regarding type 2 diabetes status (*n* = 362). Finally, 2088 participants were included in the present analyses (Additional file 1: Fig. S1).

### Laboratory analyses

Venous blood samples were collected after 12-h overnight fasting, centrifuged at 3000r/min for 15 min, aliquoted and stored at -80 °C until analyses. We tested serum TMAO, choline and betaine at baseline by high-performance liquid chromatography with online electrospray ionization tandem mass spectrometry (HPLC–MS/MS) (Agilent 6400 Series Triple Quad LCMS; CA, USA) as described previously [15]. In brief, 60 µl of either serum sample or standards was mixed with 100 μl of acetonitrile containing 10 μM of internal standards of 9-TMAO (Toronto Research Chemicals Inc, Toronto, Canada) d9-choline and d9-betaine (Sigma-Aldrich, St. Louis, USA). Then, the samples were centrifuged at 13,000 × g for 10 min to precipitate the proteins. The remaining supernatants were injected into a SiO_2_ column (2.1 mm × 100 mm, 5 μm). 30% solution A (15 mmol/L ammonium formate, pH = 3.0) and 70% solution B (acetonitrile) were used for isocratic elution with the flow rate of 0.2 mL/min. The samples were detected with mass spectrometry after elution. The intra-assay coefficients of variation were 6.0% for TMAO, 4.91% for choline and 6.21% for betaine.

Fasting glucose and insulin were measured at baseline and follow-up visits using a Roche cobas 8000 c702 automated analyzer. HbA1c was measured using high-performance liquid chromatography with the Bole D-10 Hemoglobin A1c Program on a Bole D-10 Hemoglobin Testing System. The intra-assay coefficients of variation were 2.52% for fasting glucose and 0.75% for glycated hemoglobin. HOMA-IR was calculated as serum fasting glucose (mmol/L)*serum insulin (μIU/mL)/22.5 [29]. Serum levels of total cholesterol (TC), triacylglycerol (TG), low-density lipoprotein cholesterol (LDL-C) and high-density lipoprotein cholesterol (HDL-C) were measured at baseline using a Roche cobas 8000 c702 automated analyzer. The intra-assay coefficients of variation were 2.17% for TC, 2.86% for TG, 4.67% for LDL-C and 3.47% for HDL-C. Dyslipidemia was defined as TC ≥ 6.2 mmol/L; LDL-C ≥ 4.1 mmol/L; HDL-C < 1.03 mmol/L for men, < 1.29 mmol/L for women; TG ≥ 2.3 mmol/L; or self-reported medications [30]. Serum creatinine was measured at the first follow-up visit using the enzymatic colorimetric assay (Sekisui Chemical Co., Ltd., Tokyo, Japan) with a Hitachi 7180 automatic analyzer. The intra-assay coefficient of variation was 4.43%. Estimated glomerular filtration rate (eGFR) was calculated using the Chronic Kidney Disease Epidemiology Collaboration equation [31]. According to the 2012 KDOQI Clinical Practice Guidelines, impaired renal function was defined as eGFR < 60 mL/min/1.73 m^2^, and normal or mildly decreased renal function was defined as eGFR ≥ 60 mL/min/1.73 m^2^ [32].

### Assessment of type 2 diabetes cases

Type 2 diabetes cases were ascertained at baseline (for exclusion) and follow-up visits if participants met one of the following conditions: fasting glucose ≥ 7.0 mmol/L, glycated hemoglobin ≥ 6.5%, or self-reported diabetes medications, according to the American Diabetes Association for type 2 diabetes diagnosis [33]. We ascertained 254 incident cases during a median follow-up time of 8.9 years.

### Covariate assessments

Trained investigators conducted the face-to-face interviews at baseline and each follow-up visit. We collected information on demographic characteristics (e.g., age, sex, household income and education level), lifestyles (e.g., smoking status, alcohol drinking and tea drinking), chronic disease history and medication use. Physical activity, including exercise, leisure-time activity, housework and occupation-related activity, and other daily activities, was estimated using a 19-item questionnaire and metabolic equivalent·h/d [34]. A validated 79-item food frequency questionnaire was used to estimate the habitual dietary intakes during the past year [35]. Energy intake was calculated according to the Chinese Food Composition Table 2004 [36].

Anthropometric measurements and blood pressure were measured by trained project members using the same method and equipment at baseline and each follow-up visit. When participants took off shoes and wore light clothing, height and weight were measured with an accuracy of 0.1 cm for height and 0.1 kg for weight. When participants stood erect, waist circumference and hip circumference were measured to the nearest 0.1 cm. Blood pressure was measured on the left arm with a mercury sphygmomanometer after participants had seated comfortably for at least 10 min. All indexes were measured twice, and the averages of all the readings were calculated for data analyses. Body mass index (BMI) was calculated as weight (kg) divided by the square of height (m^2^). The ratio of waist to hip circumference (WHR) was equal to waist circumference (cm) divided by hip circumference (cm). Abdominal obesity was defined as waist-hip ratio ≥ 0.90 cm for men or ≥ 0.85 cm for women [37]. Hypertension was defined as self-reported hypertension medications, systolic blood pressure (SBP) ≥ 140 mmHg or diastolic blood pressure (DBP) ≥ 90 mmHg [38].

### Statistical analysis

Serum TMAO was divided into three groups according to sex-specific tertiles: 0.63, 1.42, 3.73 μmol/L for men; 0.67, 1.37, 4.30 μmol/L for women. The differences in baseline characteristics according to tertiles of serum TMAO were examined using one-way ANOVA or Kruskal–Wallis test for continuous variables, χ^2^ test for categorical variables. Quantitative variables were presented as mean ± standard deviation (SD) or median (interquartile range, IQR), and categorical variables were presented as percentage (%).

We used Cox proportional hazards model to estimate the association between tertiles of serum TMAO and incident type 2 diabetes, taking the lowest tertile group as reference. Model 1 was adjusted for age and sex. Model 2 was adjusted for model 1 plus household income, smoking status, alcohol drinking, tea drinking, hypertension, WHR, physical activity, and intakes of total energy, egg, red and processed meat, fish and shellfish, serum levels of TG, HDL-C and fasting glucose. Hazard ratios (HRs) and corresponding 95% confidence intervals (95% CIs) were presented. Linear trends were calculated by treating the median values of serum TMAO levels in tertiles as continuous variables in the Cox regression models. We also conducted sensitivity analyses based on the final model. We repeated analyses excluding incident type 2 diabetes cases which occurred within one year after baseline or excluding those not within the mean ± 3SD of ln-transformed serum TMAO. We additionally adjusted for eGFR and exclude those with eGFR < 60 mL/min/1.73m^2^. In addition, serum choline and serum betaine (TMAO-precursor) were adjusted in the multivariable-adjusted Cox regression models. The potential non-linearity association between serum TMAO and diabetes risk was estimated with the use of a restricted cubic spline model. However, we did not find any significant non-linear association.

We performed stratified analyses in subgroups defined by several pre-defined variables: age, sex, abdominal obesity, hypertension, dyslipidemia, fasting glucose, red and processed meat intake, fish and shellfish intake, serum choline and serum betaine, to test whether the associations between tertiles of serum TMAO and type 2 diabetes risk were different. We also explored the interactions between serum TMAO and pre-defined variables by adding multiplicative terms into the multivariable-adjusted model. Prospective associations between tertiles of serum TMAO and the yearly changes in glycemic traits (serum fasting glucose, HbA1c, insulin, HOMA-IR) across time were examined by the linear mixed-effects models (LMEMs) after adjustment for potential confounding variables.

Data were inputted by Epidata 3.0 software (The EpiData Association, Odense, Denmark) and performed by STATA statistical software version 15.0 (Stata Corp., TX). A two-sided *P*-value < 0.05 was considered statistically significant.

## Results

During a median follow-up of 8.9 years (16,214 person-years), 254 incident cases of type 2 diabetes were ascertained among 2,088 participants. 72.7% of participants were women, the mean age of participants at baseline was 57.2 (SD 4.9) years, and the mean BMI was 23.0 (SD 3.0) kg/m^2^. The median (IQR) of serum TMAO was 1.54 (0.86–2.91) μmol/L. The baseline characteristics across tertiles of serum TMAO are presented in Table 1. Participants in the highest tertile of serum TMAO were more likely to be smokers, consumed more egg, fish and shellfish, had a higher prevalence of abdominal obesity and a higher level of HbA1c, compared with the lowest tertile. There were no significant differences in other baseline characteristics among the three groups of serum TMAO. Additional file 1: Table S1 presents the baseline characteristics for participants included and participants without follow-up information. Participants without follow-up information had higher BMI, SBP, DBP, serum fasting glucose and TG levels, lower income levels than participants included, but similar serum TMAO levels.

**Table 1:** Baseline characteristics according to tertiles (T) of serum TMAO

	Tertiles of serum TMAO			*P*-value
	T1 (*n* = 696)	T2 (*n* = 696)	T3 (*n* = 696)	
Age, y	56.8 ± 4.7	57.5 ± 5.1	57.4 ± 4.9	0.176
Women, %	72.7	72.7	72.7	1.000
Household income, %				0.053
≤ 1500 Yuan/Month/Person	37.4	33.5	33.3	
1501–3000 Yuan/Month/Person	43.1	49.9	45.6	
> 3000 Yuan/Month/Person	19.5	16.7	21.1	
Family history of diabetes, %	9.3	9.2	11.1	0.429
Smoker, %	12.5	11.8	16.4	0.026
Alcohol drinker, %	5.3	3.9	6.8	0.058
Tea drinker, %	48.9	48.0	50.1	0.721
Physical activity, MET-h/d	42.9 ± 16.1	42.5 ± 15.9	43.1 ± 15.5	0.904
Total energy intake, kcal/d	1858 ± 498	1822 ± 503	1817 ± 515	0.123
Red and processed meat intake, g/d	86.4 ± 45.0	85.0 ± 43.3	82.8 ± 43.3	0.371
Fish and shellfish intake, g/d	50.7 ± 52.8	51.4 ± 42.0	64.9 ± 75.0	< 0.001
Egg intake, g/d	27.3 ± 21.2	29.7 ± 19.8	29.6 ± 18.0	0.004
Dairy products intake, g/d	119.3 ± 115.1	115.0 ± 117.5	122.9 ± 115.3	0.123
BMI, kg/m^2^	22.9 ± 3.1	23.0 ± 3.0	23.3 ± 3.0	0.067
WHR	0.87 ± 0.06	0.88 ± 0.06	0.88 ± 0.06	0.108
Abdominal obesity, %	55.0	57.6	61.5	0.048
SBP, mmHg	122 ± 17	123 ± 18	122 ± 16	0.986
DBP, mmHg	78 ± 11	78 ± 11	78 ± 10	0.452
Hypertension, %	27.6	27.4	26.4	0.871
eGFR^a^, ml/min/1.73 m^2^	82.8 ± 12.1	80.5 ± 12.6	81.4 ± 12.8	0.849
Serum TMAO, μmol/L	0.66 (0.41–0.99)	1.38 (1.08–2.14)	4.04 (2.40–6.70)	< 0.001
Serum choline, μmol/L	19.1 (13.5–24.8)	18.7 (14.2–25.1)	19.1 (14.2–25.9)	0.719
Serum betaine, μmol/L	51.1 (41.5–61.1)	51.5 (42.5–63.0)	51.9 (41.1–62.0)	0.755
Serum fasting glucose, mmol/L	4.56 ± 0.61	4.61 ± 0.67	4.59 ± 0.65	0.346
Insulin, μIU/mL	8.46 ± 4.87	8.99 ± 5.32	8.87 ± 5.12	0.164
HOMA-IR	1.84 ± 1.17	1.92 ± 1.41	1.98 ± 1.44	0.503
HbA1c, %	5.52 ± 0.44	5.61 ± 0.46	5.64 ± 0.59	0.004
Serum TG, mmol/L	1.51 ± 1.06	1.53 ± 1.01	1.50 ± 0.96	0.849
Serum TC, mmol/L	5.44 ± 1.06	5.44 ± 1.04	5.41 ± 1.03	0.849
Serum HDL-C, mmol/L	1.39 ± 0.33	1.40 ± 0.34	1.37 ± 0.31	0.374
Serum LDL-C, mmol/L	3.61 ± 0.90	3.61 ± 0.92	3.62 ± 0.87	0.975
Dyslipidemia, %	56.3	57.2	55.6	0.838

*TMAO* trimethylamine-N-oxide, *MET* metabolic equivalent of task, *BMI* body mass index, *WHR* ratio of waist to hip circumference, *SBP* systolic blood pressure, *DBP* diastolic blood pressure, *HbA1c* glycated hemoglobin, *HOMA-IR* homeostatic model assessment of insulin resistance, *TG* triglycerides, *TC* total cholesterol, *LDL-C* low-density lipoprotein cholesterol, *HDL-C* high-density lipoprotein cholesterol

Mean ± SD or Median (IQR) for all continuous variables

^a^Available in 1,384 women and 525 men

The association between serum TMAO and type 2 diabetes risk is presented in Table 2. In the age- and sex-adjusted model, the HR for diabetes in the highest tertile of serum TMAO was 1.47 (95% CI: 1.08–2.01, *P*-trend = 0.033), compared with the lowest tertile. After further adjusting for baseline sociodemographic, lifestyle, dietary factors, serum TG, HDL-C and fasting glucose in the final model, a positive association between tertiles of serum TMAO and type 2 diabetes risk did not change with an HR of 1.42 (95% CI: 1.03–1.96, *P*-trend = 0.031), compared the highest tertile with the lowest tertile. The positive association between serum TMAO and risk of type 2 diabetes did not change after additional adjustment for eGFR in sensitivity analyses. Furthermore, there were no substantial difference after excluding those with eGFR < 60 mL/min/1.73m^2^, excluding incident type 2 diabetes cases that occurred within one year after baseline, or excluding those not within the mean ± 3SD of ln-transformed serum TMAO (Additional file 1: Table S2).

**Table 2:** Association of serum TMAO with type 2 diabetes risk

	Tertiles of serum TMAO			*P*-trend
	T1 (*n* = 696)	T2 (*n* = 696)	T3 (*n* = 696)	
Median, μmol/L	0.66	1.39	4.05	
Case, *n*	67	90	97	
Person years	5474	5420	5319	
Model 1^a^	1.00 (Ref)	1.32 (0.96–1.82)	1.47 (1.08–2.01)	0.033
Model 2^b^	1.00 (Ref)	1.17 (0.84–1.61)	1.42 (1.03–1.96)	0.031

*TMAO* trimethylamine-N-oxide; Ref, reference

*P* for trend was calculated by treating the median values of serum TMAO levels in tertiles as continuous values in Cox proportional hazard models

^a^Model 1: adjusted for age and sex

^b^Model 2: adjusted for model 1 plus household income, smoking status, alcohol drinking, tea drinking, hypertension, WHR, physical activity, intakes of total energy, egg, red and processed meat, fish and shellfish, serum levels of TG, HDL-C and fasting glucose

**Fig. 1:**
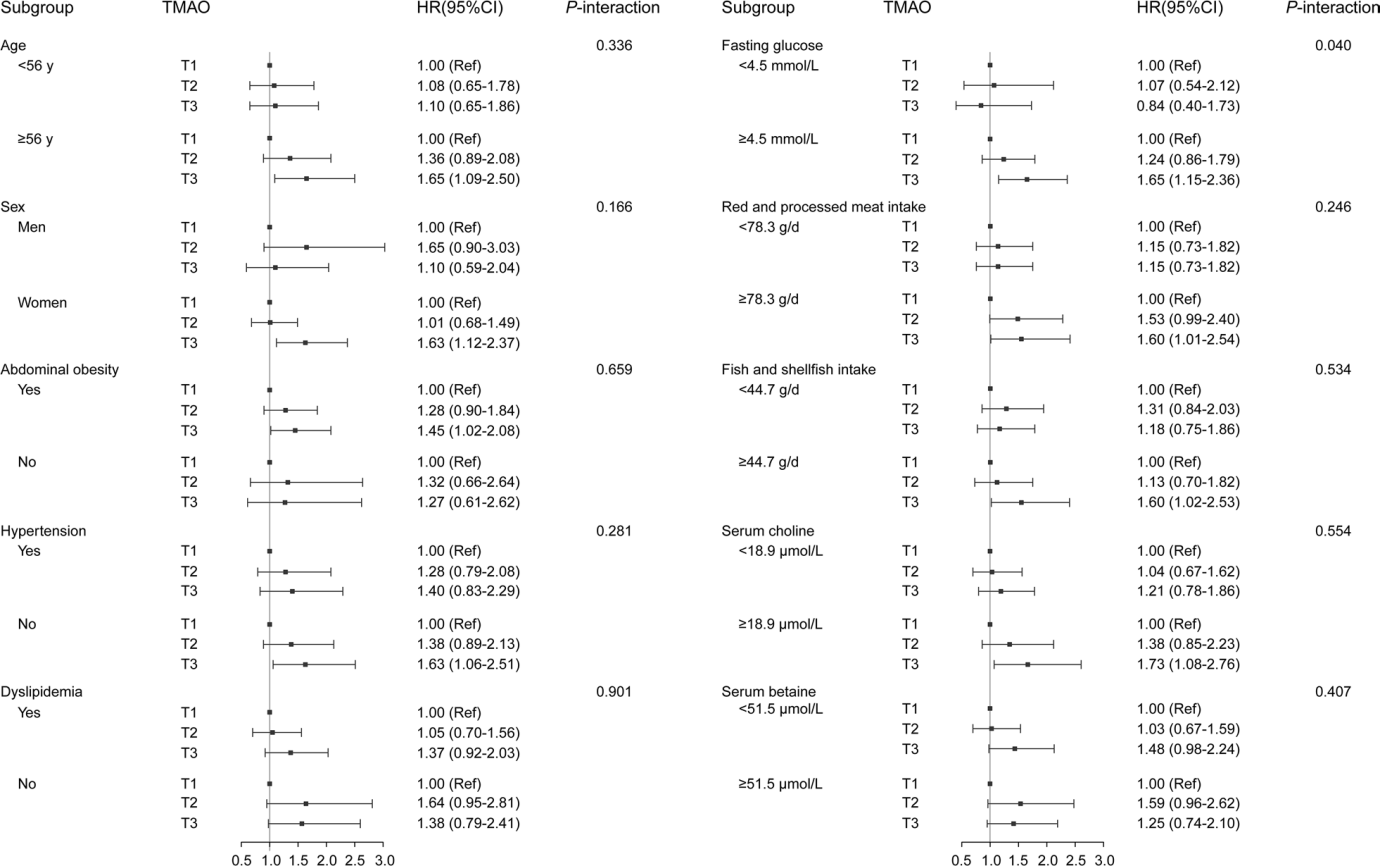
Stratified HRs and 95% CIs of type 2 diabetes according to tertiles (T) of serum TMAO^a^. *TMAO* trimethylamine-N-oxide, *HR* hazard ratio, *95% CI* 95% confidence interval, *T* tertile. ^a^In multivariable-adjusted model, confounding factors included age, sex, household income, smoking status, alcohol drinking, tea drinking, hypertension, WHR, physical activity, intakes of total energy, egg, red and processed meat, fish and shellfish, serum levels of TG, HDL-C and fasting glucose. The medians of age, serum fasting glucose, serum choline, serum betaine, red and processed meat intake, fish and shellfish intake, or egg intake were cut-off points

The stratified analyses in subgroups divided by age (< 56 [median], ≥ 56 y), sex (men, women), abdominal obesity (yes, no), hypertension (yes, no), dyslipidemia (yes, no), fasting glucose (< 4.5 [median], ≥ 4.5 mmol/L), intakes of red and processed meat (< 78.3 [median], ≥ 78.3 g/d), fish and shellfish (< 44.7 [median], ≥ 44.7 g/d), serum choline (< 18.9 [median], ≥ 18.9 μmol/L) and serum betaine (< 51.5 [median], ≥ 51.5 μmol/L), are shown in Fig. 1. There was a potential interaction between serum TMAO and baseline fasting glucose (*P*-interaction = 0.040). Compared with the first tertile of serum TMAO, HR for diabetes risk of the third tertile was 1.65 (95% CI: 1.15–2.36, *P*-trend = 0.006) among participants with a higher level of fasting glucose (≥ median), but this positive association was not observed among those with a lower level of fasting glucose (< median). There were no significant interactions between serum TMAO and other pre-defined variables.

In the LMEMs (Table 3), the estimated annual change in fasting glucose was 0.011 (0.001–0.022) mmol/L/y in the highest tertile of serum TMAO, compared with the lowest tertile (*P*-interaction = 0.044). However, no significant association was observed between serum TMAO with longitudinal changes in HbA1c, insulin or HOMA-IR.

**Table 3:** Linear mixed-effects models for association between serum TMAO and changes in glycemic traits

	Serum TMAO	
	Coefficient (95% CI)	*P*-value
Serum fasting glucose		
Time, mmol/L/y	0.089 (0.081, 0.097)	< 0.001
Group (Ref. T1)		
T2	− 0.010 (− 0.072, 0.052)	0.753
T3	− 0.022 (− 0.085, 0.040)	0.485
Time × group		
Time × T2	0.007 (− 0.003, 0.018)	0.185
Time × T3	0.011 (0.001, 0.022)	0.044
HbA1c		
Time, %/y	0.237 (0.180, 0.294)	< 0.001
Group (Ref. T1)		
T2	0.082 (− 0.565, 0.730)	0.803
T3	0.212 (− 0.440, 0.863)	0.524
Time × group		
Time × T2	0.059 (− 0.023, 0.142)	0.160
Time × T3	0.020 (− 0.063, 0.103)	0.632
Insulin		
Time, μIU/mL/y	0.130 (0.067, 0.193)	< 0.001
Group (Ref. T1)		
T2	− 0.017 (− 0.697, 0.663)	0.961
T3	− 0.149 (− 0.842, 0.543)	0.672
Time × group		
Time × T2	0.022 (− 0.068, 0.112)	0.632
Time × T3	0.073 (− 0.018, 0.165)	0.115
HOMA-IR		
Time,/y	0.076 (0.058, 0.093)	< 0.001
Group (Ref. T1)		
T2	− 0.020 (− 0.209, 0.169)	0.836
T3	− 0.039 (− 0.232, 0.153)	0.687
Time × group		
Time × T2	0.011 (− 0.015, 0.036)	0.412
Time × T3	0.022 (− 0.004, 0.048)	0.101

*TMAO* trimethylamine-N-oxide, *95% CI* 95% confidence interval, *T* tertile, *Ref* reference, *HbA1c* glycated hemoglobin, *HOMA-IR* homeostatic model assessment of insulin resistance

Linear mixed-effects models were used to estimate the association between tertile of serum TMAO and the yearly changes of glycemic traits (fasting glucose, HbA1c, insulin, HOMA-IR) overt time. All models were adjusted for baseline age, sex, household income, smoking status, alcohol drinking, tea drinking, hypertension, WHR, physical activity, intakes of total energy, egg, red meat and processed meat, fish and shellfish, serum levels of TG, HDL-C and fasting glucose

## Discussion

In this 8.9-year prospective cohort study of 2,088 middle-aged and older Chinese adults, we found that higher serum TMAO was associated with a greater risk of type 2 diabetes and an increase in fasting glucose.

Previous studies have demonstrated the positive associations between circulating concentration of TMAO and cardiovascular disease risk [16] and have suggested the adverse effects on specific cardiometabolic biomarkers, such as homocysteine, insulin and glucose [39]. However, the association between TMAO and diabetes risk remains inconsistent. Our finding was consistent with several cross-sectional studies and case–control studies, which showed a positive association [22, 23, 27], whereas others observed an inverse [25] or null association [26, 28]. A meta-analysis (including twelve clinical studies), which reported a positive association between circulating TMAO levels and diabetes risk (OR: 1.89; 95% CI: 1.63–2.19) [21]. However, most of the included studies focused on the association of TMAO and cardiovascular diseases; only four studies addressed the relationship between TMAO and diabetes. Confounding by indicators related to cardiovascular disease may remain an issue in these studies. To our knowledge, few studies were designed to investigate the prospective association between circulating TMAO and type 2 diabetes risk with fully adjustment for important lifestyle factors. In a metabolomic analysis of type 2 diabetes risk with two cohorts of Chinese adults, positive associations were observed but did not reach statistical significance [28]. However, semiquantitative measurement was used to test plasma TMAO without standard curves in that study, which was different with our study. No significant association between TMAO and incident diabetes was observed among Norwegian patients with suspected stable angina pectoris [26] or among older US adults [24]. On the contrary, a case-cohort design study within the Prevención con Dieta Mediterránea study among the elderly population at high cardiovascular disease risk in Spanish suggested that higher baseline plasma TMAO was associated with a decreased risk of type 2 diabetes [25]. Differences in genetic predisposition and dietary habits may explain the discrepancies in studies from different locations [40]. An international pooled analysis and other population-based studies have found that the associations between specific animal foods and circulating TMAO levels vary among populations [39, 41].

Circulating concentration of TMAO is affected by various factors, including diet, gut microbiota and liver flavin monooxygenase activity [9], which may modify the association between circulating TMAO and chronic disease. Fish, red meat and egg are the dominant sources of TMAO or TMAO-precursors [42]. Serum TMAO was correlated with intakes of fish and shellfish, but not red meat in this study (Table 1), which was consistent with the results from some European and Asian populations [39, 43, 44], but association of red meat with TMAO was significant in US populations [42]. We adjusted intakes of TMAO-contributing foods, including fish, red meat and egg, in the final model to reduce the potential confounding effects, and the positive association between serum TMAO and diabetes did not change. This study lacks data on gut microbiota or the activity of FMO3, we cannot explore the interaction between diet, gut microbiota and host health. On the other hand, TMAO is excreted by the kidney and renal function is a major factor influencing circulating levels of TMAO [45, 46]. A meta-analysis, including 32 eligible clinical studies, showed a negative association between circulating TMAO and renal function [47]. To reduce residual confounding of renal function, we excluded subjects with chronic renal failure. Estimated glomerular filtration rate (eGFR) plays a critically important role in signifying renal function [48]. In this study, less than 5% participants (n = 84) with impaired renal function (eGFR < 60 mL/min/1.73m^2^) were found. Moreover, we excluded subjects with eGFR < 60 mL/min/1.73m^2^ and additionally adjusted for eGFR in the sensitivity analyses, but no substantial difference was found (Additional file 1: Table S2). Given that the association between TMAO and diabetes remains inconsistent and renal function may mediate the association, more studies are needed to include eGFR and determine the interaction between TMAO, renal function and incident diabetes.

Epidemiological evidence of the association between circulating TMAO and longitudinal changes in markers of diabetes is limited. In this study, we found that serum TMAO was positively associated with an increase in fasting glucose levels. However, a longitudinal cohort study showed the null association between TMAO levels with a 2-year change in fasting glucose among 300 diabetes-free adults [49]. This negative finding might account for small sample size and short-time follow-up, which compromised the statistic power. In the future, prospective studies with large-scale and long-time follow-up are required to determine the association between circulating TMAO concentration with incident diabetes and changes in glycemic traits.

There are several possible underlying mechanisms between TMAO and diabetes. Supplemental TMAO to a high-fat diet in mice exacerbated impaired glucose tolerance, suppressed the hepatic insulin signaling pathway and increased adipose tissue inflammation, leading to insulin resistance and diabetes [50]. The gut microbiota-initiated TMA-FMO3-TMAO pathway has been identified as a contributor in the occurrence and progression of cardiometabolic diseases [51, 52]. Sudha B.Biddinger et al. found that FMO3 induced forkhead box transcription factor O1 (FoxO1, a key driver of metabolic disease) by producing TMAO. TMAO bound and activated the endoplasmic reticulum stress kinase PERK (a key sensor of intracellular stress), and then PERK induced FoxO1, which promoted insulin resistance and metabolic dysfunction [11]. Furthermore, manipulation of gut microbiota or knockdown of FMO3 in insulin-resistant mice inhibited TMAO production, reduced PERK activation and suppressed FoxO1 in the liver, which may prevent the development of hyperglycemia [11, 51]. On the other hand, our previous study has reported that TMAO could modulate bile acid metabolism and suppress bile acids-mediated hepatic nuclear receptor farnesoid X receptor (FXR) signaling to aggravate hepatic steatosis [53]. Bile acids modulation and FXR pathway also regulate glucose metabolism to cause obesity and diabetes [54]. However, that TMAO may affect bile acids and FXR to induce diabetes has not been reported. Further studies are warranted to explore potential mechanisms of TMAO and diabetes pathogenesis.

The strengths of this study include its prospective and population-based design, over 8.9-year follow-up period and a high rate of follow-up (> 85%). Besides, we adjusted a variety of known diabetes risk factors in statistical models to reduce potential confounding effects. However, this study has several limitations. Firstly, participants were middle-aged and older adults in southern China, and our findings were limited to generalize to other age groups or populations. Secondly, we only measured serum TMAO at baseline. Changes in serum TMAO over time could not be included in data analyses. Thirdly, we did not conduct an oral glucose tolerance test to ascertain type 2 diabetes, which may lead to undiagnosed diabetes cases. Fourthly, our study lacked data regarding the activity of FMO3 and gut microbiota, which were determining circulating TMAO level.

## Conclusions

Our study suggested that higher serum TMAO was associated with increased type 2 diabetes risk and an increase in fasting glucose among middle-aged and older adults. More studies are needed to identify the role of TMAO on the pathogenesis and progression of diabetes.

## Supplementary Information

**Additional file 1:** **Figure S1.** Flow chart of the study participants. **Table S1. **Baseline characteristics of participants included and participants without follow-up information. **Table S2.** Sensitivity analyses for association between serum TMAO and incident type 2 diabetes.

## Data Availability

The datasets used and/or analyzed during the current study are available from the corresponding author on reasonable request.

## Abbreviations

TMAO: Trimethylamine-N-oxide
GNHS: The Guangzhou Nutrition and Health Study
HR: Hazard ratio
CI: Confidence interval
IQR: Interquartile range
TMA: Trimethylamine
FMO3: Flavin-containing monooxygenase-3
HPLC–MS/MS: High-performance liquid chromatography with online electrospray ionization tandem mass spectrometry
TC: Total cholesterol
TG: Triacylglycerol
LDL-C: Low-density lipoprotein cholesterol
HDL-C: High-density lipoprotein cholesterol
BMI: Body mass index
WHR: Ratio of waist to hip circumference
SBP: Systolic blood pressure
DBP: Diastolic blood pressure
SD: Standard deviation
eGFR: Estimated glomerular filtration rate
PERK: Protein kinase R-like endoplasmic reticulum kinase
FoxO1: Forkhead box transcription factor O1
FXR: Farnesoid X receptor
