# Supplementary material for: Serum trimethylamine-N-oxide is associated with incident type 2 diabetes in middle-aged and older adults: a prospective cohort study
Source: J Transl Med. 2022 Aug 18;20:374. doi: 10.1186/s12967-022-03581-7 (PMC9389664; doi:10.1186/s12967-022-03581-7)
Supplement: Supplementary file 1 — Additional file 1: Figure S1. Flow chart of the study participants. Table S1. Baseline characteristics of participants included and participants without follow-up information. Table S2. Sensitivity analyses for association between serum TMAO and incident type 2 diabetes. [file 12967_2022_3581_MOESM1_ESM.docx]

**Additional file 1 materials**


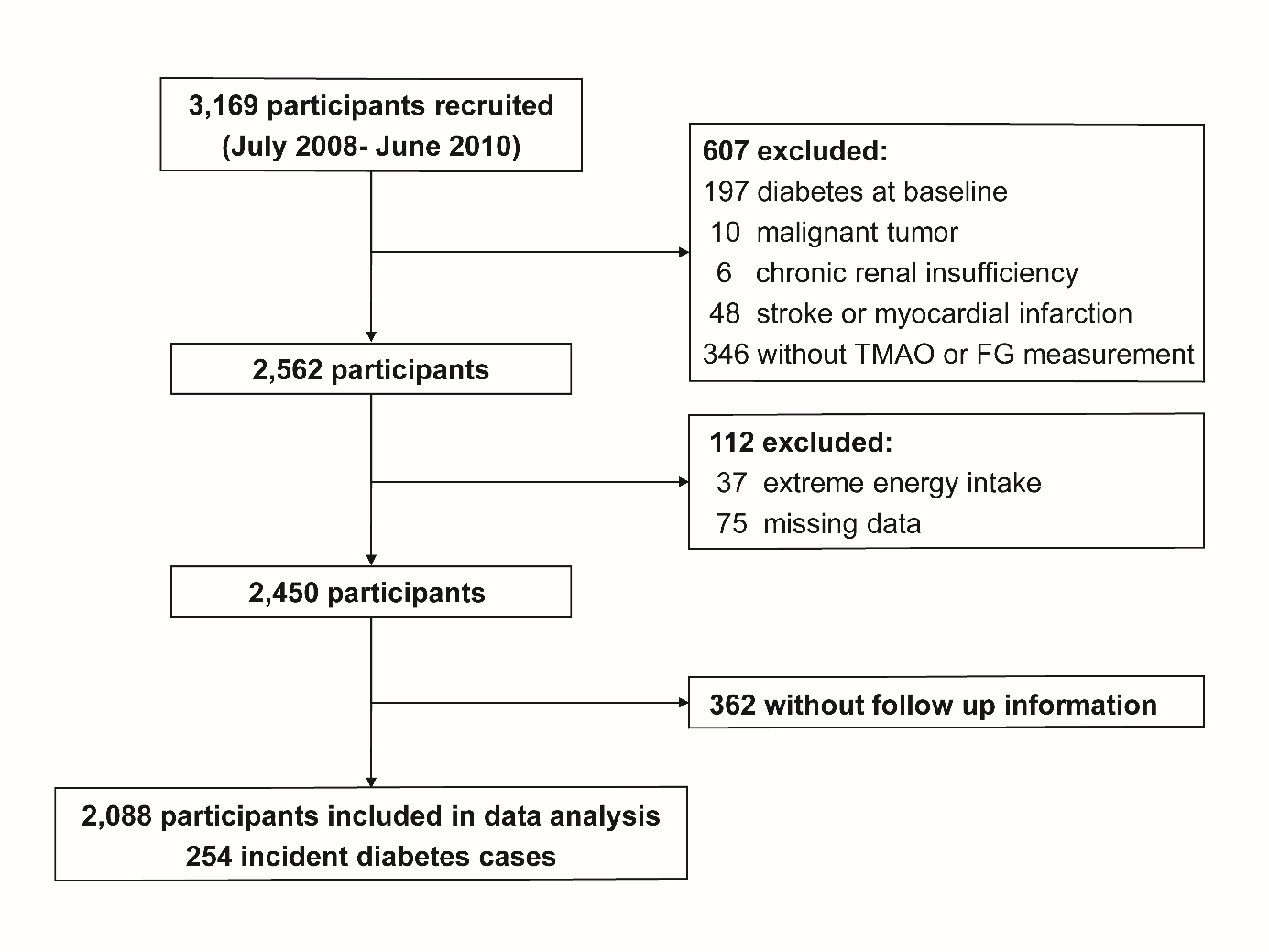
 **Figure S1.** Flow chart of the study participants

TMAO, trimethylamine-N-oxide; FG, fasting glucose.

**Table S1.** Baseline characteristics of participants included and participants without follow-up information ^a^

|  | Participants included (*n*=2,088) | Participants without follow-up information (*n*=362) | *p*-value |
| --- | --- | --- | --- |
| Age, y | 57.2 ± 4.9 | 57.5 ± 5.6 | 0.320 |
| Women, % | 72.7 | 70.7 | 0.436 |
| Household income, % |  |  | **0.006** |
| ≤1500 Yuan/Month/Person | 34.7 | 43.4 |  |
| 1501-3000 Yuan/Month/Person | 46.2 | 38.9 |  |
| >3000 Yuan/Month/Person | 19.1 | 17.7 |  |
| Smoker, % | 14.0 | 16.6 | 0.126 |
| Alcohol drinker, % | 5.3 | 5.8 | 0.706 |
| Tea drinker, % | 49.0 | 51.7 | 0.349 |
| Physical activity, MET-h/d | 42.8 ± 15.8 | 42.2 ± 16.0 | 0.458 |
| Total energy intake, kcal/d | 1832 ± 506 | 1822 ± 533 | 0.512 |
| Red and processed meat intake, g/d | 84.6 ± 44.4 | 84.5 ± 43.3 | 0.744 |
| Fish and shellfish intake, g/d | 55.6 ± 58.3 | 52.4 ± 46.0 | 0.702 |
| Egg intake, g/d | 28.9 ± 19.8 | 30.1 ± 19.6 | 0.282 |
| Dairy products intake, g/d | 118.8 ± 115.7 | 115.8 ± 123.0 | 0.153 |
| BMI, kg/m^2^ | 23.0 ± 3.0 | 23.5 ± 3.4 | **0.006** |
| WHR | 0.88 ± 0.06 | 0.89 ± 0.07 | **0.007** |
| SBP, mmHg | 122 ± 17 | 126 ± 19 | **<0.001** |
| DBP, mmHg | 78 ± 11 | 79 ± 11 | **0.004** |
| Serum TMAO, μmol/L | 1.54 (0.86-2.91) | 1.50 (0.98-2.88) | 0.657 |
| Serum choline, μmol/L | 18.9 (14.0-25.2) | 18.7 (13.9-25.0) | 0.561 |
| Serum betaine, μmol/L | 51.5 (41.8-62.0) | 50.9 (41.7-64.6) | 0.524 |
| Serum fasting glucose, mmol/L | 4.59 ± 0.64 | 4.78 ± 0.77 | **<0.001** |
| Serum TG, mmol/L | 1.51 ± 1.01 | 1.63 ± 1.07 | **0.027** |
| Serum TC, mmol/L | 5.43 ± 1.05 | 5.47 ± 1.11 | 0.598 |
| Serum HDL-C, mmol/L | 1.38 ± 0.33 | 1.37 ± 0.33 | 0.236 |
| Serum LDL-C, mmol/L | 3.62 ± 0.90 | 3.70 ± 0.94 | 0.105 |

Abbreviations: MET, metabolic equivalent of task; BMI, body mass index; WHR, ratio of waist to hip circumference; SBP, systolic blood pressure; DBP, diastolic blood pressure; TMAO, trimethylamine-N-oxide; FG, fasting glucose; TG, triglycerides; TC, total cholesterol; LDL-C, low-density lipoprotein cholesterol; HDL-C, high-density lipoprotein cholesterol.

^a^ Mean ± SD or Median (IQR) for all continuous [variable](file:///C:\Users\john\AppData\Local\Youdao\Dict\Application\7.5.0.0\resultui\dict\?keyword=variable)s.

**Table S2.** Sensitivity analyses for association between serum TMAO and incident type 2 diabetes

| Sensitivity analyses | Tertiles of serum TMAO | | | *p*-trend ^a^ |
| --- | --- | --- | --- | --- |
|  | T1 | T2 | T3 |  |
| Model 2 ^b^ | 1.00 (Ref) | 1.18 (0.85-1.62) | 1.46 (1.06-2.00) | **0.021** |
| Model 2a ^c^ | 1.00 (Ref) | 1.25 (0.90-1.73) | 1.44 (1.04-1.99) | **0.042** |
| Model 2b ^d^ | 1.00 (Ref) | 1.18 (0.85-1.62) | 1.42 (1.04-1.95) | **0.033** |
| Model 2c ^e^ | 1.00 (Ref) | 1.08 (0.77-1.50) | 1.48 (1.08-2.05) | **0.008** |
| Model 2d ^f^ | 1.00 (Ref) | 1.16 (0.83-1.63) | 1.51 (1.08-2.10) | **0.032** |
| Model 2e ^g^ | 1.00 (Ref) | 1.18 (0.86-1.63) | 1.44 (1.05-1.98) | **0.026** |
| Model 2f ^h^ | 1.00 (Ref) | 1.20 (0.87-1.66) | 1.43 (1.04-1.97) | **0.033** |

Abbreviations: TMAO, trimethylamine-N-oxide; Ref, reference.

^a^ *P* for trend was calculated by treating the median values of serum TMAO levels in tertiles as continuous values in Cox proportional hazard models.

^b^ Model 2: adjusted for age, sex, household income, smoking status, alcohol drinking, tea drinking, hypertension, WHR, physical activity, intakes of total energy, egg, red and processed meat, fish and shellfish, serum levels of TG, HDL-C and fasting glucose.

^c^ Model 2a: excluding type 2 diabetes cases occurring within one year after baseline (*n*=6).

^d^ Model 2b: excluding those not within the mean±3SD of ln-transformed serum TMAO (*n*=8).

^e^ Model 2c: adjusted for Model 2 plus eGFR.

^f^ Model 2d: excluding those with eGFR < 60 mL/min/1.73m^2^ (*n*=84).

^g^ Model 2e: adjusted for Model 2 plus serum choline.

^h^ Model 2f: adjusted for Model 2 plus serum betaine.
